# Supplementary material for: Biochemical Characterization of Emerging SARS-CoV-2 Nsp15 Endoribonuclease Variants
Source: bioRxiv. 2022 May 12:2022.05.10.491349. Preprint. [Version 1] doi: 10.1101/2022.05.10.491349 (PMC9128782; doi:10.1101/2022.05.10.491349)
Supplement: 1 [file NIHPP2022.05.10.491349v1-supplement-1.pdf]

bioRxiv preprint doi: https://doi.org/10.1101/2022.05.10.491349; this version posted May 12, 2022. The copyright holder for this preprint (which was not certified by peer review) is the author/funder. This article is a US Government work. It is not subject to copyright under 17 USC 105 and is also made available for use under a CC0 license.

Supplemental Information:

Supplemental Tables 1-2
Supplemental Figures 1-5
Supplemental Files 1-5

Legend for Supplemental Files

Supplemental File 1. Original GISAID download and analysis, June 15, 2021. See ReadMe tab in spreadsheet for additional information.

Supplemental File 2. Feb 20, 2022 GISAID download and analysis. See ReadMe tab in spreadsheet for additional information.

Supplemental File 3. Identification of the earliest date of origin of each of the non-synonymous Nsp15 substitutions.

GISAID accession IDs for all Nsp15 proteins that contain each substitution of interest were used to query all of the fasta headers for each Nsp15 protein in GISAID downloaded on 02/15/2022. Each worksheet is a compilation of these headers for a specific substitution of interest, sorted by the oldest date. Each column in each worksheet is a different element in each of the fasta headers for these sequences. The column headers are left in the GISAID format dictated by their upload webpage (https://www.epicov.org/epi3/frontend#5045a9). Column YYY-MM-DD is the collection date for each genome and is the only date we can attach to these proteins. Based on these dates we can estimate the date and location of each of these substitutions. As some of the dates are incomplete, we are basing the date of origin only on dates that were completely provided by submitters.

Supplemental File 4. Raw data for the data in Supplemental Table 2.

Supplemental File 5. Table acknowledging the GISAID contributions of submitting and originating laboratories.

bioRxiv preprint doi: https://doi.org/10.1101/2022.05.10.491349; this version posted May 12, 2022. The copyright holder for this preprint (which was not certified by peer review) is the author/funder. This article is a US Government work. It is not subject to copyright under 17 USC 105 and is also made available for use under a CC0 license.

Construct	Mutation Location (domain level)	First created
WT-Nsp15 (6xHis-thrombin-TEV/pet14-b)	N/A	P[28]
Nsp15 K13N	NTD	This study
Nsp15 K13R	NTD	This study
Nsp15 G18E	NTD	This study
Nsp15 G18R	NTD	This study
Nsp15 T34I	NTD	This study
Nsp15 A93S	MD	This study
Nsp15 T115A	MD	This study
Nsp15 V128F	MD	This study
Nsp15 D133Y	MD	This study
Nsp15 L163F	MD	This study
Nsp15 P206S	EndoU	This study
Nsp15 R207S	EndoU	This study
Nsp15 D220Y	EndoU	This study
Nsp15 H235Y	EndoU	This study
Nsp15 K260R	EndoU	This study
Nsp15 K290N	EndoU	This study
Nsp15 W333C	EndoU	This study

Supplemental Table 1: Plasmid Constructs used in this study. All constructs generated by Genscript (Piscataway, NJ).

bioRxiv preprint doi: https://doi.org/10.1101/2022.05.10.491349; this version posted May 12, 2022. The copyright holder for this preprint (which was not certified by peer review) is the author/funder. This article is a US Government work. It is not subject to copyright under 17 USC 105 and is also made available for use under a CC0 license.

Residue Substitution	Alleles	All	Omicron	Delta	Delta/Omicron
K13R	AG	0.00022722	0.00000725	0.00004861	6.703142054
K13N	GC	0.00000381	0.00000242	0.00000351	1.450199002
K13N	GT	0.00080130	0.00006816	0.00121778	17.86521749
G18R	GA	0.00037332	0.00001257	0.00069664	55.42363109
G18R	GC	0.00000229	0.00000048	0.00000397	8.217794345
G18E	GA	0.00030400	0.00002395	0.00058364	24.37424752
T34I	CT	0.00177838	0.00011589	0.00114276	9.860321698
A93S	GT	0.00015042	0.00000287	0.00005678	19.75894765
T115A	AG	0.00086410	0.00001006	0.00136916	136.1404271
V128F	GT	0.00172299	0.00108040	0.00160257	1.483305117
D133Y	GT	0.00083478	0.00001772	0.00125023	70.5573099
L163F	CT	0.00127986	0.00036301	0.00186168	5.128482846
P206S	CT	0.00202981	0.00029213	0.00199766	6.838251464
R207S	GC	0.00000152	0.00000000	0.00000187	NA
R207S	GT	0.00095044	0.00002251	0.00053294	23.67752014
D220Y	GT	0.00174677	0.00004023	0.00081121	20.16551036
H235Y	CT	0.00242754	0.00061970	0.00391448	6.316740301
K260R	AG	0.00298775	0.00002586	0.00427359	165.2541891
K290N	.C	0.00000022	0.00000000	0.00000000	NA
K290N	.T	0.00000033	0.00000000	0.00000000	NA
K290N	GC	0.00000163	0.00000048	0.00000210	4.350597006
K290N	GT	0.00018909	0.00005555	0.00023411	4.214232768
W333C	GC	0.00000033	0.00000000	0.00000023	NA
W333C	GT	0.00027989	0.00001772	0.00047780	26.96499696

Supplemental Table 2: The frequency of occurrence of each non-synonymous Nsp15 substitution in the Delta and Omicron variants of concern. GISAID accession IDs for each of the genomes containing a substitution listed in the Residue Substitution column were used to search the Custom Download page (https://www.epicov.org/epi3/frontend#4fa597) using the Select function. Prior to the search, the "Variant" selector was used to limit the search to either VOC Omicron, VOC Delta, or all variants. All is the frequency of each substitution in all SARS CoV2 genomes in GISAID; Delta is the frequency of each substitution in the Delta subset of SARS CoV2 genomes; Omicron is the frequency of each substitution in the Omicron subset of SARS CoV2 genomes. Delta/Omicron is the ratio of the count of each substitution in each of the two VOC. For raw data see Supplemental File Four.

bioRxiv preprint doi: https://doi.org/10.1101/2022.05.10.491349; this version posted May 12, 2022. The copyright holder for this preprint (which was not certified by peer review) is the author/funder. This article is a US Government work. It is not subject to copyright under 17 USC 105 and is also made available for use under a CC0 license.

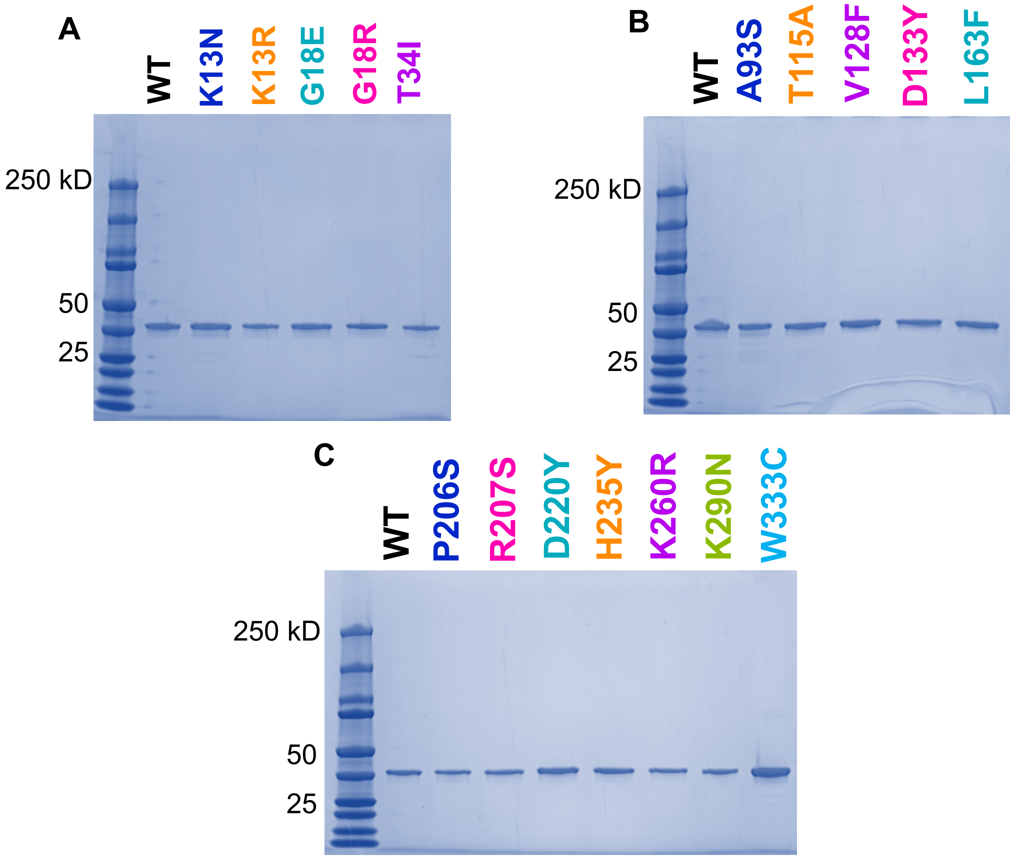

Supplemental Figure 1: Summary gels following purification of all Nsp15 variants. Fractions corresponding to active hexamer were analyzed by SDS-PAGE and reveal pure protein for NTD **(A)**, MD **(B)**, and EndoU **(C)** variants.

bioRxiv preprint doi: https://doi.org/10.1101/2022.05.10.491349; this version posted May 12, 2022. The copyright holder for this preprint (which was not certified by peer review) is the author/funder. This article is a US Government work. It is not subject to copyright under 17 USC 105 and is also made available for use under a CC0 license.

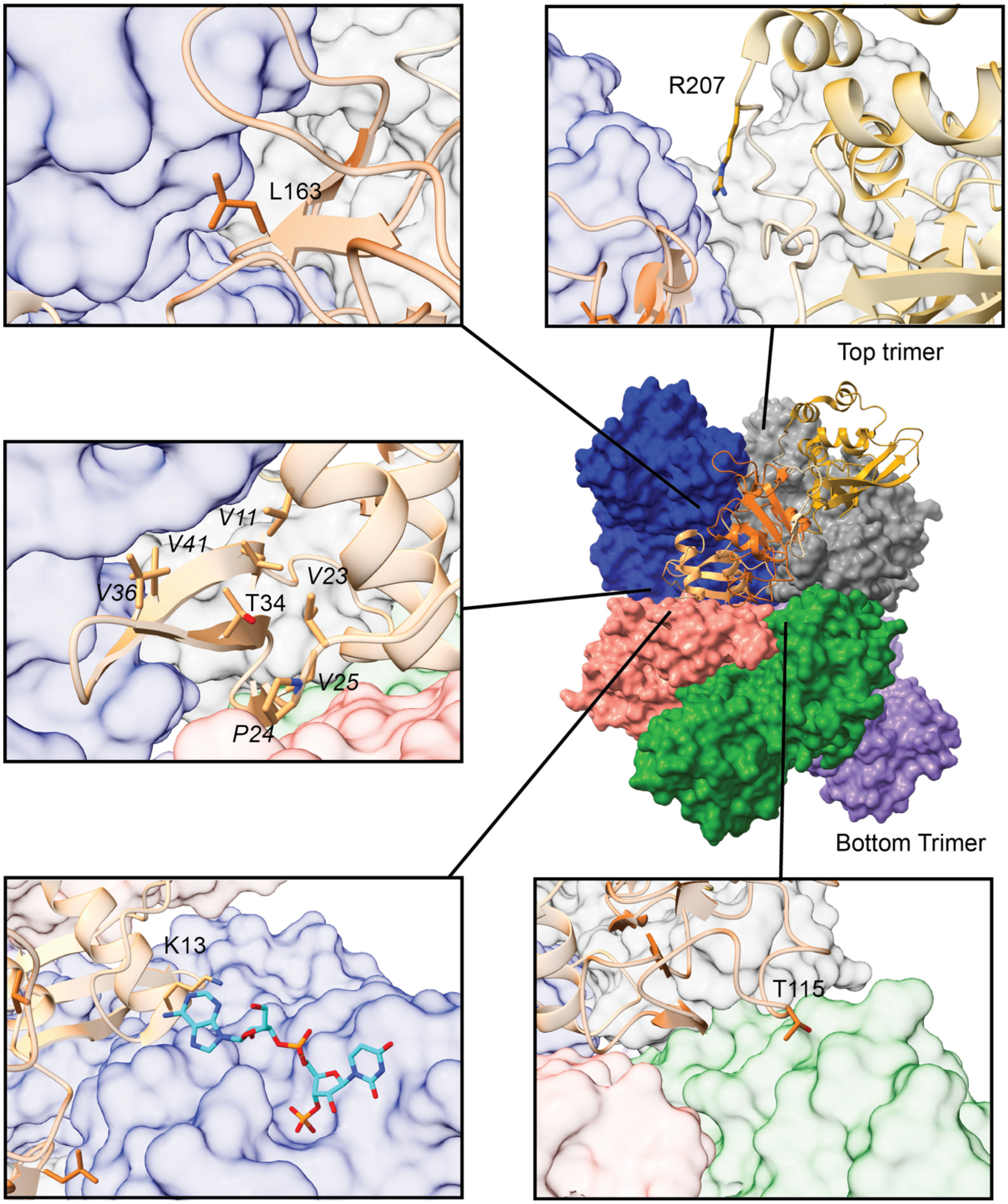

Supplemental Figure 2: Selected residues involved in hexamer interface interactions. Center right: Nsp15 hexamer (PDB: 7N06). Five protomers shown in surface view (blue, grey, salmon, green, purple); one protomer shown in ribbon view and colored by domain (NTD, tan; MD, orange; EndoU, gold). Boxes depict zoomed in regions with residues of interest. From top right, counter-clockwise: EndoU residue R207, MD residue L163, NTD residue T34 with surrounding core residues labeled in italics, NTD residue K13, and MD residue T115. K13 is shown with a post-cleavage RNA; the 5' base extends towards the neighboring NTD including K13.

bioRxiv preprint doi: https://doi.org/10.1101/2022.05.10.491349; this version posted May 12, 2022. The copyright holder for this preprint (which was not certified by peer review) is the author/funder. This article is a US Government work. It is not subject to copyright under 17 USC 105 and is also made available for use under a CC0 license.

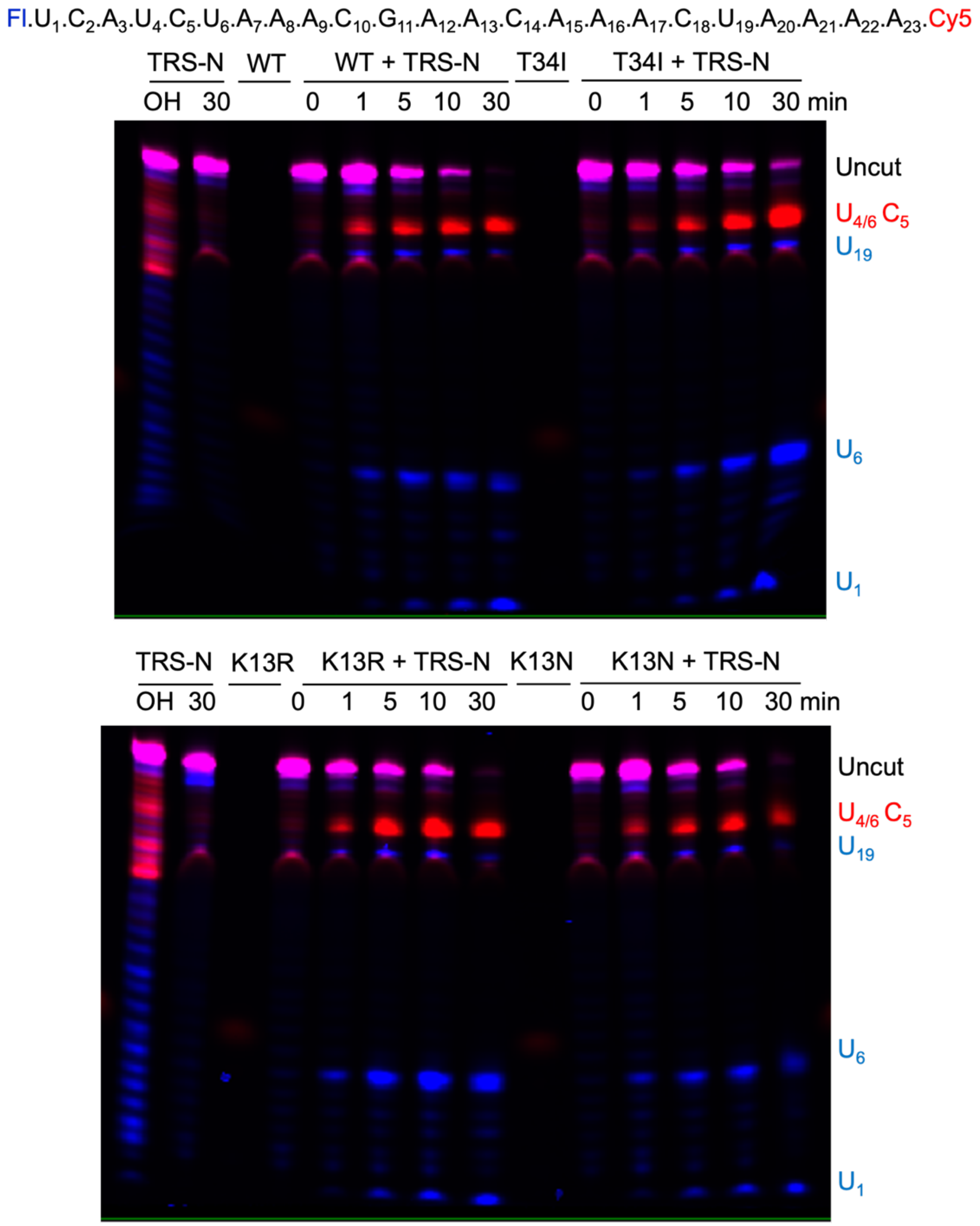

bioRxiv preprint doi: https://doi.org/10.1101/2022.05.10.491349; this version posted May 12, 2022. The copyright holder for this preprint (which was not certified by peer review) is the author/funder. This article is a US Government work. It is not subject to copyright under 17 USC 105 and is also made available for use under a CC0 license.

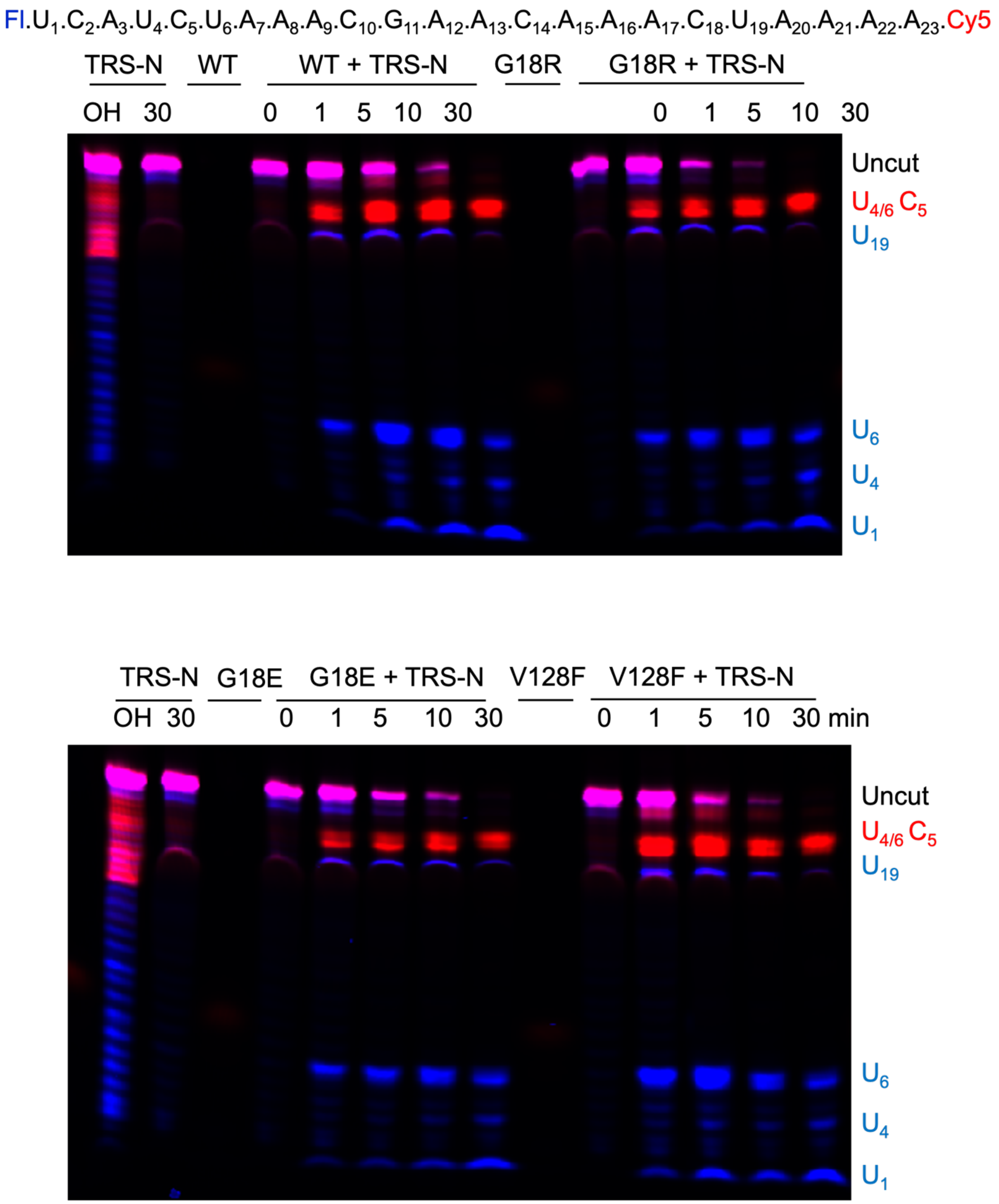

Supplemental Figure 3: Gel-based endonuclease assays for NTD/MD mutants. The transcriptional regulatory sequence for the nucleocapsid protein (TRS-N) is fluorescently labeled on both end (see labeled sequence at top of each set of gels). A 30-min time course nuclease assay was carried out with WT or Nsp15 NTD/MD variants.

bioRxiv preprint doi: https://doi.org/10.1101/2022.05.10.491349; this version posted May 12, 2022. The copyright holder for this preprint (which was not certified by peer review) is the author/funder. This article is a US Government work. It is not subject to copyright under 17 USC 105 and is also made available for use under a CC0 license.

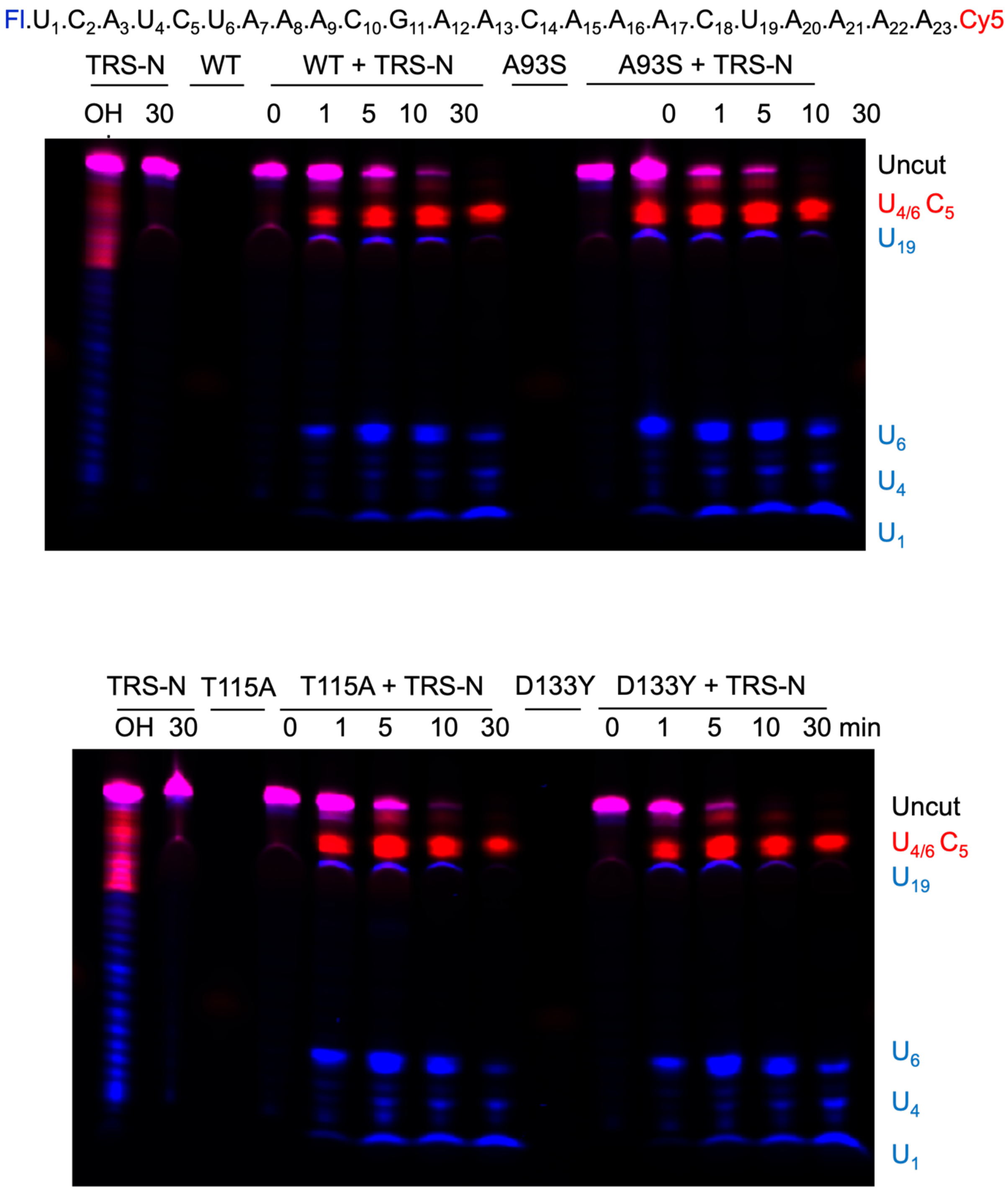

bioRxiv preprint doi: https://doi.org/10.1101/2022.05.10.491349; this version posted May 12, 2022. The copyright holder for this preprint (which was not certified by peer review) is the author/funder. This article is a US Government work. It is not subject to copyright under 17 USC 105 and is also made available for use under a CC0 license.

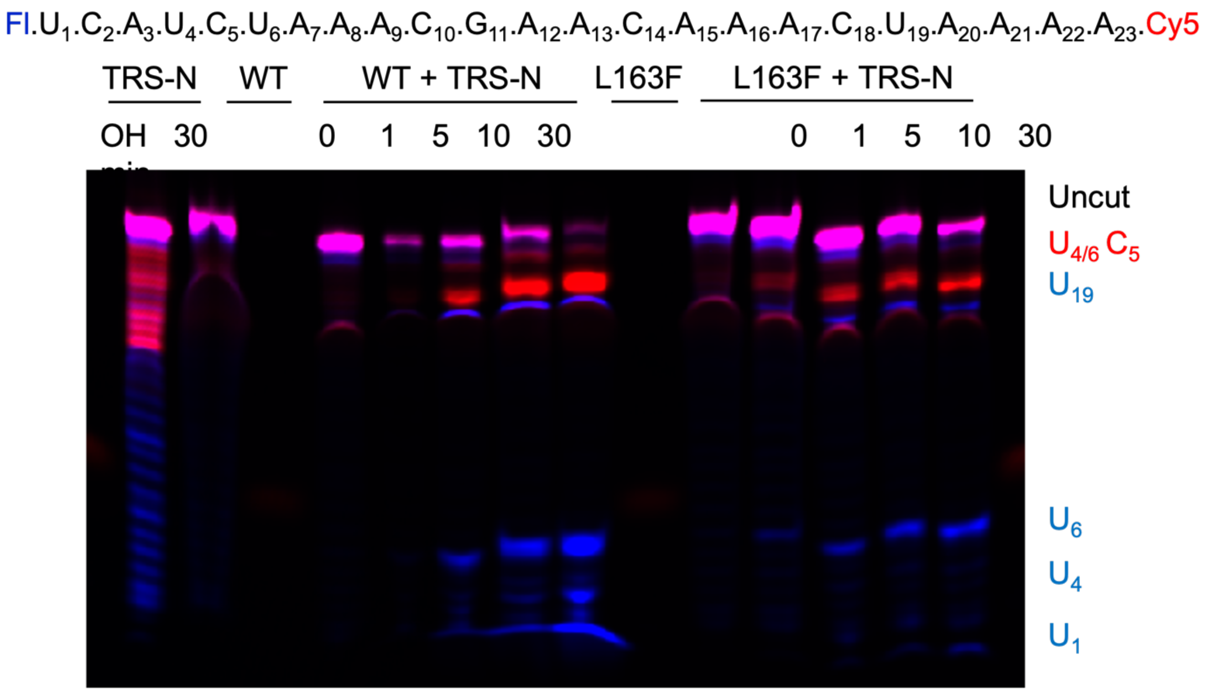

Supplemental Figure 4: Gel-based endonuclease assays for MD mutants. The transcriptional regulatory sequence for the nucleocapsid protein (TRS-N) is fluorescently labeled on both end (see labeled sequence at top of each set of gels). A 30-min time course nuclease assay was carried out with WT or Nsp15 MD variants.

bioRxiv preprint doi: https://doi.org/10.1101/2022.05.10.491349; this version posted May 12, 2022. The copyright holder for this preprint (which was not certified by peer review) is the author/funder. This article is a US Government work. It is not subject to copyright under 17 USC 105 and is also made available for use under a CC0 license.

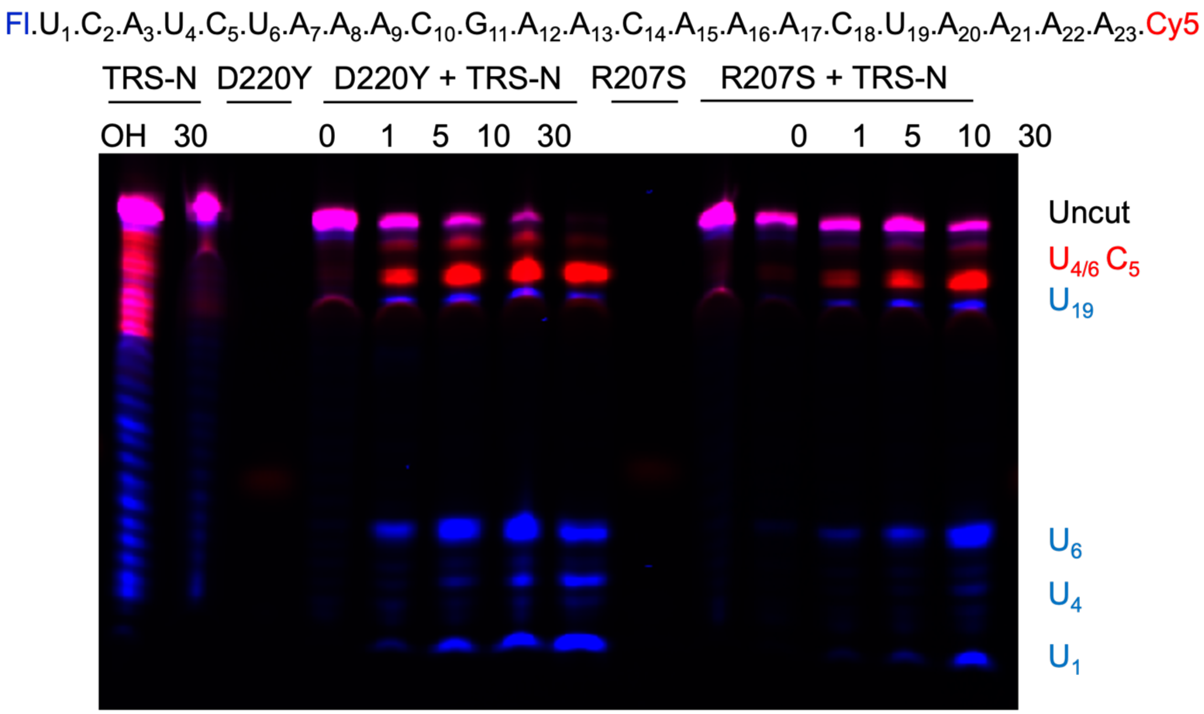

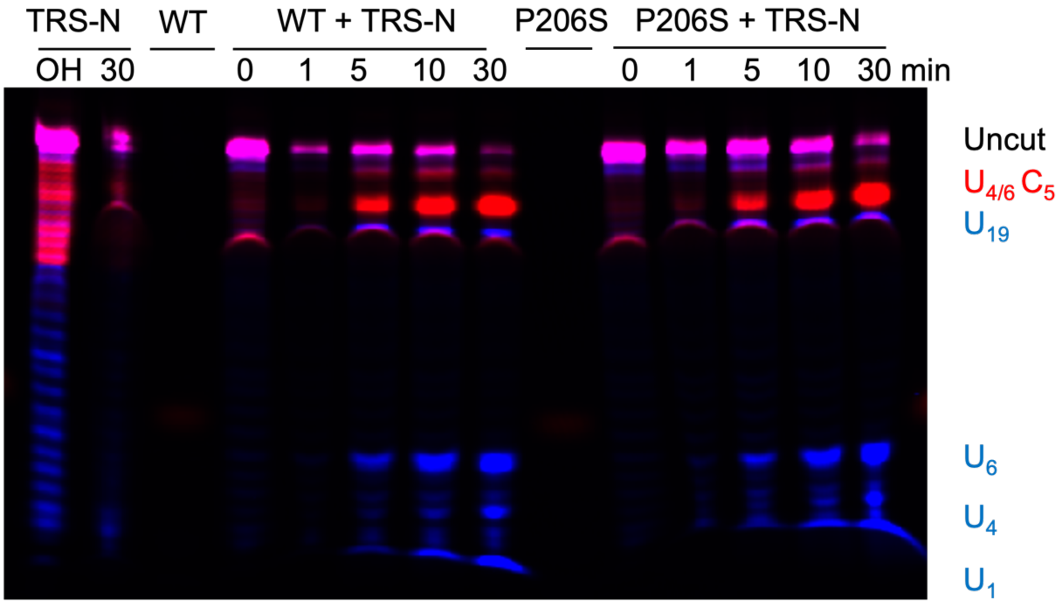

bioRxiv preprint doi: https://doi.org/10.1101/2022.05.10.491349; this version posted May 12, 2022. The copyright holder for this preprint (which was not certified by peer review) is the author/funder. This article is a US Government work. It is not subject to copyright under 17 USC 105 and is also made available for use under a CC0 license.

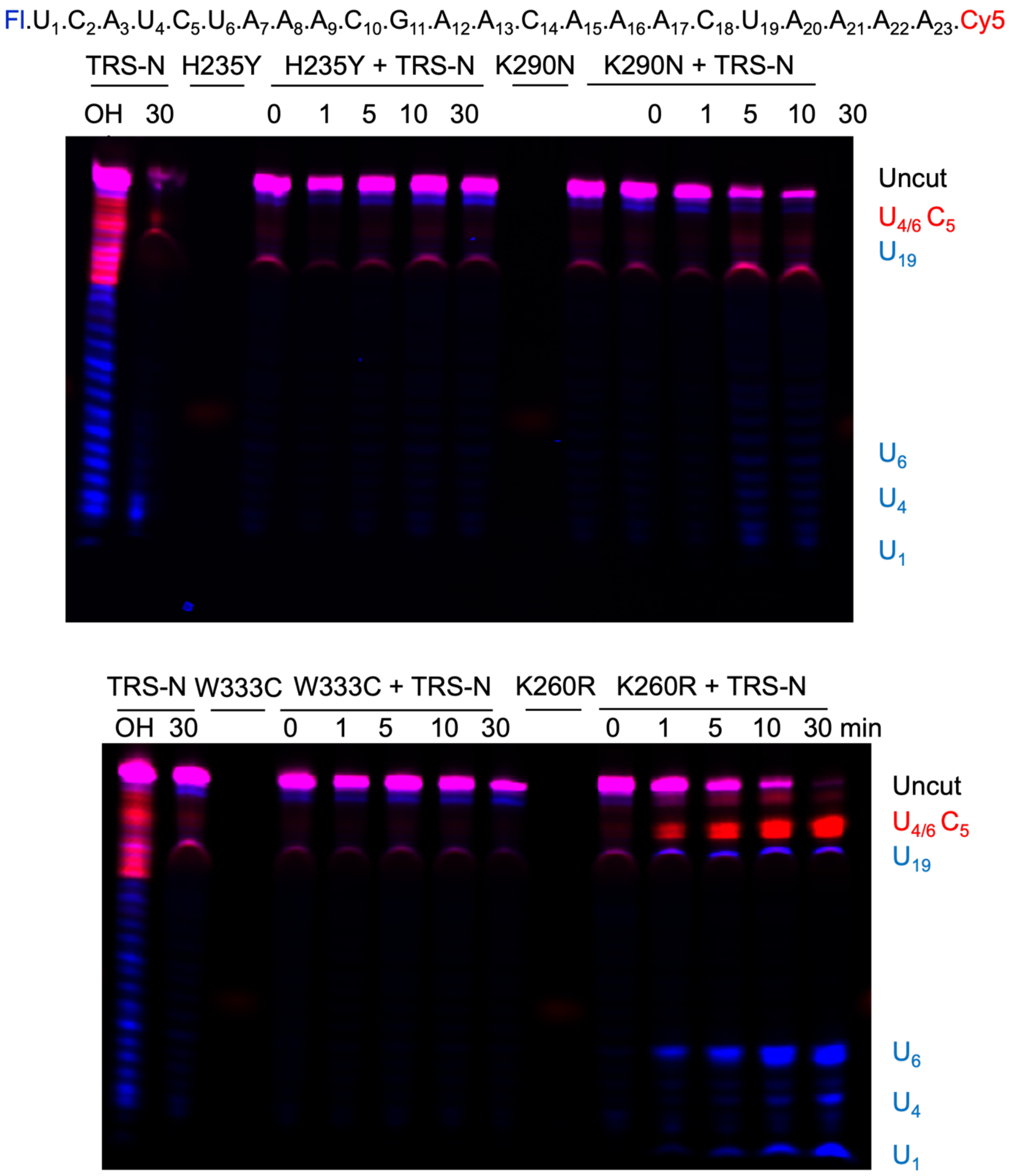

Supplemental Figure 5: Gel-based endonuclease assays for EndoU mutants. The transcriptional regulatory sequence for the nucleocapsid protein (TRS-N) is fluorescently labeled on both end (see labeled sequence at top of each set of gels). A 30-min time course nuclease assay was carried out with WT or Nsp15 EndoU variants.
